# Early Life Professional and Layperson Support Reduce Poor Oral Hygiene Habits in Toddlers—A Prospective Birth Cohort Study

**DOI:** 10.3390/dj6040056

**Published:** 2018-10-08

**Authors:** Diep Hong Ha, Loc Giang Do

**Affiliations:** Australian Research Centre for Population Oral Health, Adelaide Dental School, The University of Adelaide, Adelaide 5005, Australia; diep.ha@adelaide.edu.au

**Keywords:** oral health behaviours, early life, dental advice, lay support, birth cohort

## Abstract

Oral health behaviours of children are formulated from a very young age. Formation of those behaviours among very young children is dependent on their mothers/caregivers who may themselves require support from the health profession or laypersons. The study aimed to investigate if early life visits for check-up and dental advice and perceived support improved oral health behaviours as practiced by mothers of toddlers aged 24–30 months old. Data from a population-base birth cohort study in South Australia was used. The study recruited and followed mothers of newborn children from birth to age 24–30 months. Parental questionnaires collected information about socioeconomic factors, dental visiting patterns, and oral health behaviours as practiced by the mothers for their child. Self-reported putting a child to bed with a bottle and brushing a child’s teeth were the outcome variables. The two main exposures of this study were (1) early visiting for a dental advice, and (2) layperson support that a mother received in the first two years of having the child. Data were analysed progressively from bivariate to multivariable regression models. A total of 1183 mother/child dyads had complete data. The retained sample was representative of the population. Approximately 36% of mothers put their child to bed with a bottle and 26% of mothers did not brush their child’s teeth the night before. Around 29% of children had a visit for dental check-up and 80% of mothers reported having lay support. There were gradients in the outcome variables by socioeconomic factors and the main exposures. Multivariable regression models reported that having no dental visit for advice and having no lay support were associated with 1.30 and 1.21 imes higher rates of putting a child to bed with a bottle, respectively. Having no dental visit for advice was associated with a 1.37-times higher rate of not brushing a child’s teeth, controlling for other factors. This population-based birth cohort study confirmed importance of early life dental visit for check-up and support for mothers of young children in establishing oral health behaviours of young children.

## 1. Introduction

Early childhood caries (ECC) in children is one of the most prevalent chronic diseases in childhood. For children, ECC is defined as having any decay on any teeth rather than smooth surfaces and severe early childhood caries (S-ECC), is defined as any sign of smooth surface caries (cavitated or not) in children younger than three years of age [1]. Risk factors for childhood caries are multifactorial ranging from upstream community-level determinants to family-level influences and individual factors [2]. Dental care system and social support belong to community- and family-level influences. Downstream individual factors include microbiota (determined by oral hygiene status) and dietary patterns, which can be influenced by unfavourable dental health behaviours, such as infrequent toothbrushing and having a bottle before bed. Maintaining the primary dentition in a healthy condition is important for the well-being of the child. The primary dentition is required for proper mastication, aesthetics, phonetics, space maintenance, and for prevention of aberrant habits. Reducing dental plaque formation by brushing a child’s teeth, and modification of dietary habits such as unfavourable feeding behaviour (nocturnal feeding) are essential for the prevention of ECC and maintaining oral health for life.

Despite significant improvement in living conditions and dental services, the prevalence and severity of dental caries in preschool children remained high. The number of decayed, missing, or filled primary teeth (dmft) among 4-year-old Australian children fluctuated from 1.10 in 1997 to 1.44 in 1999, to 1.70 in 2003–2004, and 1.84 in 2005 [3,4]. A study reported a prevalence of ECC of 5.6% examined at 20 months of age among South Australian children [5]. S-ECC also affects a child’s general health, learning ability and future oral health [6,7]. Yet, S-ECC is largely preventable by targeting risk factors accountable for its development. 

Poor oral hygiene practice is a risk factor for early childhood caries. Its effect has been evidenced in both cross-sectional and longitudinal studies [8,9]. A systematic review concluded that the effect of infrequent brushing on incidence and increment of carious lesions was higher in the primary (with and odds ratios (OR) of 1.75 and 95% confidence interval (CI) from 1.49 to 2.06) than in the permanent dentition [10]. Hence, frequent toothbrushing is needed since early life. Toothbrushing is a main approach to reducing or removing plaque, especially before going to bed. Toothbrushing is also a means to deliver frequent exposure to fluoride. This oral health practice should be done by mother/caregiver for infants and very young children [11,12]. Toothbrushing without toothpaste is recommended from the time of tooth eruption. Toothbrushing with fluoridated toothpaste should then follow when children are able to spit out. The Australian Guidelines on Fluoride Use recommend commencement of toothbrushing with fluoridated toothpaste during the age from 18 to 30 months [13]. Hence, the role of parents or caregivers in this oral health practice is important for young children of that age group [14]. Nevertheless, a significant proportion of children did not start toothbrushing with toothpaste after the age of 30 months [14]. One in five Australian children aged 5–6 did not brush their teeth at least twice a day [14].

There is a belief among mothers/caregivers that bottle feeding helps a toddler to sleep. Hence, a bottle before bed is often used as a soothing method, considered as safe by most mothers/caregivers. However, putting children to sleep with bottle containing milk or a sweetened drink is a known risk factor for ECC [15,16]. Sweetened drinks before bed were found to be associated with risk of *S Mutans* colonization in very young children [17]. A longitudinal cohort study found that sleeping with a bottle at 30 months was associated with increased risk of ECC [18]. Another study found that children who nursed freely from bottle at night beyond their eighth month of life had almost twice as many dental caries as children who did not [19].

The American Academy of Paediatric Dentistry recommends an initial oral evaluation visit within six months of the eruption of the first primary tooth, but not later than 12 months of age. As evidence of effectiveness for that recommendation was weak [20], the infant oral health care visit should be seen as the foundation upon which a lifetime of preventive education and dental care can be built to ensure optimal oral health [21].

Young children in South Australia can visit a wide range of healthcare providers for dental advice, including early childhood nurses, general healthcare providers, oral health therapists, and dentists. However, use of such early preventive dental advice is a source of persisting oral health inequality [22]. On the other hand, studies have also proved that preventive dental care such as anticipatory guidance for parents was effective in reducing caries incidence among their young children [23,24]. While the recommendation for early dental visits exists, however, much of the evidence has been from cross-sectional studies. It is important to further understand the causal effect of early oral health advice on unfavourable dental health behaviours. This knowledge will improve the evidence base for advice to parents/caregivers of very young children.

Mothers of young children often need support from people around them to provide proper care for their young children [12,25]. Availability of lay support may vary across socioeconomic groups. Hence, this factor may influence variations in unfavourable dental health behaviours leading to different levels of risk for dental caries since early life. The possible effect of lay support for mothers in caring for young children deserve evaluation using longitudinal cohort data.

**Figure 1:**
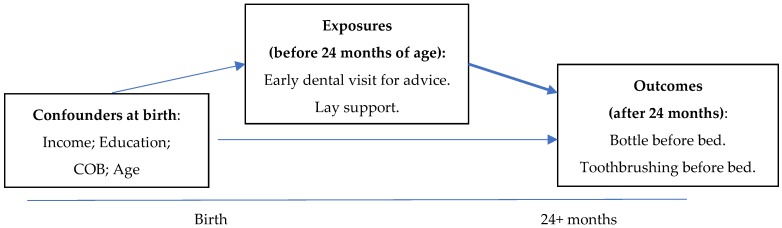
Schematic design of the study.

The aim of our study was to evaluate the effect of early visit(s) for oral health advice during the first two years of life and the availability of lay support to mothers in caring for young children on unfavourable dental health behaviours for the children using data from a population-based longitudinal birth cohort study. It was hypothesised that background confounders at the birth of the child determined the exposures, which in turn influenced the primary outcomes when children became older (See Figure 1).

## 2. Methods

This study analysed data collected from the Study of Mothers and Infants Life Events Affecting Oral Health (SMILE), a South Australian population-based birth cohort study of socio-economically diverse children [26]. Mother–infant dyads were recruited from three main metropolitan public hospitals in Adelaide, South Australia between July 2013 and August 2014. Recruitment typically took place within the first 48 hours after birth by trained health professionals who provided mothers with a written and verbal description of the study. The mothers were recruited consecutively and all newborns, regardless of birth weight and gestational age, were eligible to participate. Women whose English was insufficient to comprehend the written and verbal instructions and those living outside of the greater Adelaide area, or intending to relocate within the next 12 months, were excluded. We deliberately attempted to over-recruit women from low socio-economics areas because of higher attrition rate expected for this group. Mothers of newborn children were invited to participate and complete enrolment details and a questionnaire at birth. A total of 2112 mother–infant dyads had completed enrolment and baseline (wave 1) questionnaire. Participants were subsequently contacted when their children turned 3 (wave 2), 6 (wave 3), 12 (wave 4), and 24 months (wave 5) to complete subsequent follow-up questionnaires. The baseline questionnaire data were collected through face-to-face interview at the time of recruitment. The follow-up questionnaires were administered through online, paper-based, or telephone interviews. Participating women were contacted with different means of communication in order to achieve a high level of response.

Ethical approval for SMILE was obtained from the Southern Adelaide Clinical Human Research Ethics Committee (HREC# 50.13, approval date 28 February 2013) and the South Australian Women and Children Health Network (HREC# 13/WCHN/69, approval date 7 August 2013). Women were informed that their participation was voluntary and informed consent was obtained from all mothers who participated in the study.

Participants who completed the questionnaire when their child had turned 24 months of age (wave 5) were included in this study. The primary outcomes (hereafter referred as put child to bed with bottle and not brushing child’s teeth before bed) was assessed via the questionnaire. The questionnaire asked about frequency of a child being put to bed with a bottle. If a child was reported as being usually put to bed with a bottle at most bedtimes, and the content of the bottle was not water, the child was considered as having this unfavourable behaviour. The wave 5 questionnaire also asked if children’s teeth were brushed before bed the day before completion of the survey. If a child’s teeth were not brushed before bedtime then the child was categorised as not brushing teeth before bed. The primary outcomes were used as binary variables in the analysis.

The two main explanatory variables were also collected via the wave 5 questionnaire. Visiting for dental advice was defined using three questions. Mothers were asked if their child had been seen by a healthcare professional (including child and youth health nurse, school dental service therapist, dentist, or general medical practice) and about child’s teeth/gums prior to the age of 24 months. The former two were the most commonly types of service used by mothers of young children. Those who responded positively to that question were asked about reasons of the visit. Options included check-up/oral hygiene instruction and dental problems including pain. Early dental visit was defined as having a dental visit for check-up or oral hygiene instruction within the first two year of life. The wave 4 questionnaire at the age of 12 months asked mothers about available lay support using a Likert scale; the statement was “There is a special person who is around when I am in need?”. Answering “agree” or “strongly agree” was categorised as having support when needed, “strongly disagree”, “disagree”, or “not sure” options were grouped as not having support when needed.

Potential early life socioeconomic factors and covariates were selected *a priori*. Household income was collected at birth. Annual before-tax income was assessed by the question ‘Which category does your household income fall into?’ The response options were categorised into approximate three groups: Q1: <AU$80,000; Q2: AU$80,000 to <AU$120,000; and Q3: >AU$120,000+. Potential factors collected at the baseline (birth) and subsequent interviews included the mother’s age at the child’s birth (≤24 years; 25–34 years; 35+ years), the mother’s Indigenous status, country of birth, and education attainment. Mothers were categorised as Indigenous if they classified themselves as Aboriginal or Torres Strait Islander or both. Four categories of countries of birth were created (Australia, New Zealand, and UK; India; Asia other; or all other countries). Mothers’ educational attainment was combined into having school/vocational training, and some or complete university education.

The questions used to collect information on confounders, exposures, and primary outcomes are in the supplementary materials.

We analysed data progressively from bivariate to multivariable regression models. Generalised regression models were used to estimate association between the exposure variables (early visit for dental advice and having lay support), and each of the primary outcomes (putting child to bed with a bottle and not brushing before bed) using a log-Poisson link function with robust error estimation to estimate adjusted prevalence ratio (PR) and associated 95% confidence intervals (95% CI). The models were built based on priori assumptions of associations between the confounders, the exposure variables, and the outcomes. The models also included the child’s age in months, calculated when the questionnaire used to assess the outcomes was completed. Interactions between covariates were not included in the models. All data analyses were conducted using SAS version 9.4.

## 3. Results

Data from a total of 1183 children, whose mothers completed the wave 5 questionnaire, were included in the study, representing 56% of the initial sample (Table 1). The retention rate was higher among high income participants than that among low income participants. However, the income distribution of the retained sample was comparable with the population benchmarks. The proportion of retained sample with school only or vocational training was lower than the population benchmarks. Other main characteristics were comparable with the population. Some 74.8% of mothers reported that they were born in Australia, New Zealand, or UK. Just over half of parents had university education.

Almost 29.3% of children reportedly had an early dental visit for check-up or advice about teeth and gums before the age of two years (Table 1). Most of those visits for dental advice were made at school dental service clinics and Child and Youth health centers. Approximately 84% of mothers reported that they had layperson support available during the first two years of their young child’s life.

Some 36% of children were often put to bed with bottle by mothers/care givers containing either milk/formula/fruit juice (Table 1). Some 25.6% of children had not had their teeth brushed before bed the night before completing the wave 5 questionnaire. 

In a bivariate analysis (Table 2), the percentage of mothers who put their infants to bed with a bottle were lowest among the highest income category. There was a gradient in the percentage of mothers who did not brush their child’s teeth before bed, with the highest percentage among lowest income mothers and the lowest percentage among highest income mothers. 

Mothers who reported not having an early visit for dental advice for their child were more likely to put their child to bed with a bottle than mothers who took their child for an early dental visit for advice (37.5% vs. 29.4%). There was also a significant difference in the percentage of mothers who did not brush their child’s teeth.

Mothers who had reported no lay support when they needed were more likely to put a child in bed with a bottle compared to mothers who had support (40.2% vs. 33.6%). Also, those same mothers were less likely to brush their child’s teeth before bed (30.7% vs. 23.4%). 

The effect of having an early dental visit for advice and having lay support when needed on putting child to bed with a bottle were estimated in a multivariable model adjusting for other factors (Table 3). Having no early visit for dental advice was associated with 1.3 times the rate of putting the child to bed with bottle. Having no lay support when needed was associated with an adjusted PR of 1.2 of having this unfavourable oral health behaviour. However, this effect was borderline non-significant after adjusting for other factors.

The same model also confirmed the effect of mothers’ country of birth and mothers’ education on putting a child to bed with a bottle (Table 3). Mothers who were born in India and mothers without tertiary education were more likely to put their child to bed with a bottle.

Factors affecting toothbrushing before bed were evaluated in another multivariable model (Table 3). Having no early dental visit for advice was associated with a higher rate of not brushing child’s teeth before bed, adjusting for other factors. Having no lay support when needed was associated with a higher but not significant prevalence ratio of not brushing teeth before bed.

## 4. Discussion

To the best of our knowledge, this is one of the first analyses to examine the effect of early dental visits for check-up and oral health advice on reducing mothers’ unfavourable behaviours to their young children using prospective cohort data. Very young children are dependent on their mothers/primary caregivers. Therefore, it is critical that early favourable dental health behaviours are properly carried out by their mothers/caregivers. Such behaviours in early life can potentially change trajectory of their oral health later in life.

Through early dental visit for check-up and advice before dental problems have initiated, parents have opportunity to receive oral health information, which may increase awareness of proper oral hygiene behaviours and practices. New parents may not be aware of guidelines on oral hygiene behaviours and practice such as toothbrushing [27]. A discussion with a child and youth health nurse or school dental service therapist has been proved useful to improve mothers’ oral health behaviours toward their children in our study. The results of our study were consistent with previous studies in that targeting parents of young children, especially mothers, was beneficial in the prevention of ECC [28,29].

The study results were in line with literature reported that an oral health promotion programme based on anticipatory guidance initiated during the mother’s pregnancy was successful in reducing the incidence of S-ECC in young children in South Australia (4). An early discussion with healthcare professional in this study showed a positive effect in establishing good oral health behaviours for young children. Also, an early discussion with healthcare professional about a child’s gums/teeth can potentially improve oral health literacy within the family.

The strength of this study lies in its prospective study design and population-based study sample. The evidence generated from the study is indicative of causal relationship between the explanatory variables and the outcomes. There was a limitation of the study as it relied on self-reporting by the mothers. However, systematic bias of such self-reporting was not expected.

The longer term benefit from an early discussion with mothers about child oral health by a healthcare professional needs to be explore. While this study did not directly examine the effect of the professional and layperson support on incidence of early childhood caries, two major family-level risk factors for ECC were investigated. An early visit about an infant’s teeth/gums could levelled out the inequality in mother’s unfavourable health behaviours to their child. It may lead to breaking inter-generational transfer of unfavourable behaviours from mothers to young children.

The study supports provision of dental health information to mothers and caregivers of very young children. It also supports integration of dental health and general health programs conducted by a wider range of health professionals in providing dental health information to mothers of young children.

## Figures and Tables

**Table 1:** Descriptive statistics of the study population.

Variables	Total (n = 1183) *	%	95% CI	Sample at Birth n = 2112 (%)	Population ^+^
OUTCOMES					
Put child to bed with bottle					
No	741	63.4	60.7–66.2	--	--
Yes	427	36.6	33.8–39.3	--	--
Brushing child’s teeth before bed					
No	299	25.6	23.1–28.1	--	--
Yes	868	74.4	71.9–76.9	--	--
EXPLANATORY VARIABLES					
Early dental visit for advice					
No	749	70.7	67.9–73.4	--	--
Yes	311	29.3	26.6-32.1	--	--
Lay support					
Yes	840	81.6	79.2–83.9	--	--
No	190	18.5	16.1–20.8	--	--
COVARIATES					
Family income at birth					
Q1 (<AU$80,000)	539	46.1	43.1–49.0	53.9	43.8
Q2 (AU$80,000–120,000)	353	30.2	27.5–32.9	27.6	31.8
Q3 (>AU120,000)	278	23.7	21.3–26.3	18.5	24.4
Mother’s COB					
Other	73	6.2	4.9–7.6	6.7	8.2
Asia except India	132	11.3	9.5–13.1	11.4	8.8
India	90	7.7	6.2–9.2	8.9	4.0
Aust., NZ, UK	875	74.8	72.3–77.3	73.0	79.0
Mother’s age groups					
<25 year old	124	10.7	8.9–12.5	16.3	17.8
25–34	787	68.1	65.5–70.9	64.2	62.4
>=35 year old	244	21.1	18.7–23.4	19.5	19.8
Mother’s education					
School/Vocational	626	54.1	51.2–56.9	54.0	59.2
Tertiary	532	45.9	43.1–48.8	46.0	40.8

* Some variables have missing value. n: Number of children included in this study. COB: countries of birth. 95% CI: 95% confidence interval of %. ^+^ Population distribution. -- Not available.

**Table 2:** Bivariate associations of unfavourable dental health behaviour with exposures and covariates.

Variables	Put Child to Bed with a Bottle	Did Not Brush Child’s Teeth before Bed
	% (95% CI)	% (95% CI)
**Early dental visit for advice**		
Yes	29.4 (24.3–34.4)	20.9 (16.4–25.4)
No	37.5 (34.0–41.0)	27.3 (24.1–30.5)
**Lay support**		
Yes	33.6 (30.4–36.8)	23.4 (20.5–26.3)
No	40.2 (33.2–47.2)	30.7 (24.1–37.3)
**Family income at birth**		
Q1 (<AU$80,000)	38.7 (34.6–42.9)	29.2 (25.3–33.1)
Q2 (AU$80,000–120,000)	39.8 (34.7–45.0)	24.4 (19.9–28.9)
Q3 (>AU120,000)	27.7 (22.4–32.9)	20.0 (15.3–24.7)
**Mother’s COB**		
Other	35.6 (24.6–46.6)	30.1 (19.6–40.7)
Asia except India	42.4 (34.0–50.9)	25.2 (17.8–32.6)
India	44.4 (34.2–54.7)	58.4 (48.2–68.7)
Aust., NZ, UK	35.1 (31.9–38.3)	21.9 (19.2–24.7)
**Mother’s age group**		
<25 years old	47.1 (38.2–56.0)	24.6 (17.0–32.2)
25–34 years old	35.4 (32.1–38.8)	24.4 (21.4–27.4)
≥35 years old	35.4 (29.4–41.4)	31.0 (25.2–36.8)
**Mother’s education**		
School/vocational	40.7 (36.4−44.9)	26.0 (22.2−29.8)
Tertiary	33.5 (29.9−37.1)	25.2 (21.9−28.6)

95% CI: 95% confidence intervals.

**Table 3:** Factors associated with the unfavourable oral health behaviours.

Variables	Putting Child to Bed with a Bottle		Did Not Brush Child’s Teeth before Bed	
	Adj. PR	95% CI	Adj. PR	95% CI
**Early dental visit for advice**				
Yes	Ref		Ref	
No	1.30	1.04–1.64	1.37	1.02–1.84
**Lay support**				
Yes	Ref		Ref	
No	1.21	0.97–1.49	1.20	0.93–1.55
**Family income**				
Q1 (<AU$80,000)	1.15	0.87–1.51	0.99	0.73–1.34
Q2 (AU$80,000–120,000)	1.30	0.99–1.70	1.02	0.74–1.41
Q3 (>AU120,000)	Ref		Ref	
**Mother’s COB**				
Others	1.08	0.72–1.62	1.17	0.73–1.85
Asia except India	1.44	1.07–1.93	1.26	0.86–1.83
India	1.63	1.20–2.21	2.69	1.99–3.62
Aust., NZ, UK	Ref		Ref	
**Mother’s age group**				
<25 year old	1.26	0.91–1.76	0.61	0.38–1.00
25–34	0.99	0.78–1.24	0.63	0.49–0.81
>=35 year old	Ref		Ref	
**Mother’s education**				
School/vocational	1.26	1.02–1.56	1.25	0.97–1.60
Tertiary	Ref		Ref	

Adjusted PR estimated with multivariable regression model with robust standard error estimation.

## Supplementary Materials

The following are available online at http://www.mdpi.com/2304-6767/6/4/56/s1, Questionnaire S1: Extract from the SMILE questionnaires.
