# Supplementary material for: Early Life Professional and Layperson Support Reduce Poor Oral Hygiene Habits in Toddlers—A Prospective Birth Cohort Study
Source: Dent J (Basel). 2018 Oct 8;6(4):56. doi: 10.3390/dj6040056 (PMC6313388; doi:10.3390/dj6040056)
Supplement: Supplementary file 1 [file dentistry-06-00056-s001.pdf]

Appendix: Extract from the SMILE questionnaires
Wave 1 Baseline questionnaire:

Confounding factors: SES

E7 **Please complete the table below.**

If your child lives in a <u>one-parent household</u>, please fill in one column for yourself (Mother).

If your child lives in a <u>two-parent household</u>, please fill in a column for yourself (Mother) and a column for your partner (Other Parent /Guardian).

QUESTIONS	↓ Mother	↓ Other Parent/Guardian
**a) What is <u>your</u> age?**	…………… Years Old	…………… Years Old
**b) What is your sex?** *(Tick one box only)*	☐$_1$ Male<br>☐$_2$ Female	☐$_1$ Male<br>☐$_2$ Female
**c) In which country were you born?**	☐$_1$ Australia<br>☐$_2$ Other country *(please specify)*<br>…………………………………	☐$_1$ Australia<br>☐$_2$ Other country *(please specify)*<br>…………………………………
**d) Is English your first language?**	☐$_1$ Yes<br>☐$_2$ No<br>*Please specify what other language(s) you speak at home?*<br>…………………………………....	☐$_1$ Yes<br>☐$_2$ No<br>*Please specify what other language(s) you speak at home?*<br>…………………………………....
**e) Are you of Aboriginal or Torres Strait Islander origin?** *(Tick one box only)*	☐$_1$ No<br>☐$_2$ Yes, Aboriginal<br>☐$_3$ Yes, Torres Strait Islander<br>☐$_4$ Yes, Aboriginal & Torres Strait Islander	☐$_1$ No<br>☐$_2$ Yes, Aboriginal<br>☐$_3$ Yes, Torres Strait Islander<br>☐$_4$ Yes, Aboriginal & Torres Strait Islander
**f) What is the highest level of education you have?** *(Tick one box only)*	☐$_1$ Some high school<br>☐$_2$ Completed high school<br>☐$_3$ Some vocational training (i.e. trade)<br>☐$_4$ Completed vocational training<br>☐$_5$ Some University or College<br>☐$_6$ Completed University or College<br>☐$_7$ Postgraduate	☐$_1$ Some high school<br>☐$_2$ Completed high school<br>☐$_3$ Some vocational training (i.e. trade)<br>☐$_4$ Completed vocational training<br>☐$_5$ Some University or College<br>☐$_6$ Completed University or College<br>☐$_7$ Postgraduate
**g) What is your current work status?** *(Tick one box only)*	☐$_1$ Full time employed prior to this birth<br>☐$_2$ Part time employed prior to this birth<br>☐$_3$ Unemployed *(please go to E8)*<br>☐$_4$ Home duties *(please go to E8)*<br>☐$_5$ Pensioner	☐$_1$ Full time employed<br>☐$_2$ Part time employed<br>☐$_3$ Unemployed *(please go to E8)*<br>☐$_4$ Home duties *(please go to E8)*<br>☐$_5$ Pensioner
**h) What is your main occupation?**	☐$_1$ Manager / Administrator<br>☐$_2$ Professional<br>☐$_3$ Para-professional / Trade person<br>☐$_4$ Clerk / Salesperson /Personal service worker<br>☐$_5$ Plant / Machine operator / Drivers<br>☐$_6$ Manual worker / Labourer<br>☐$_7$ Other ………………………	☐$_1$ Manager / Administrator<br>☐$_2$ Professional<br>☐$_3$ Para-professional / Trade person<br>☐$_4$ Clerk / Salesperson /Personal Service worker<br>☐$_5$ Plant / Machine operator / Drivers<br>☐$_6$ Manual worker / Labourer<br>☐$_7$ Other ………………………

E8 **Which category does your total household income (before tax) fall into?** Include any salaries, pensions, allowances, benefits, etc from all persons in the household. *(Please tick one box only)*

Household income per year

☐1 Up to $20,000	☐6 $100,001 to $120,000
☐2 $20,001 to $40,000	☐7 $120,001 to $140,000
☐3 $40,001 to $60,000	☐8 $140,001 to $160,000
☐4 $60,001 to $80,000	☐9 $160,001 to $180,000
☐5 $80,001 to $100,000	☐10 Over $180,000

Wave 4 questionnaire (age 12 months)

Exposure variable: Lay support

B32. These statements relate to the support you get from other people.

(Please tick one box only for each statement)	**Strongly disagree**				**Strongly agree**
There is a special person who is around when I am in need.	☐1	☐2	☐3	☐4	☐5

Wave 5 questionnaire (age of 24 months)

Exposure variable: Visit for dental advice

A12 **How many times has YOUR CHILD been seen by a healthcare professional about his/her teeth and/or gums in the last 12 months?** *(This includes a dentist, dental therapist, GP or nurse)*

…………… times The child has not been seen about his/her teeth ☐1 ⟶ ***PLEASE GO TO A15***

A13 **Who did YOUR CHILD see at the last dental visit?**

Private Dentist ☐1 Child and Youth Health nurse ☐4

School Dental Service dentist/therapist ☐2 Other ☐5

General Practitioner (GP) ☐3 *(Please specify)*……………………………………..

A14 **What was the reason for the visit?**

Check-up /Oral hygiene instruction ☐1 Other ☐3

Dental pain ☐2 *(Please specify)* ………………………………………

Outcome variable: Brushing before bed

PART A: YOUR CHILD'S DENTAL BEHAVIOURS AND PRACTICES

In this section we would like you to answer some questions about your child's teeth and mouth.

A1 **Were YOUR CHILD's teeth brushed before bed yesterday?**

Yes ☐1 No ☐2

Outcome variable: Putting to bed with a bottle

E2 **How often do you put YOUR CHILD to bed with a bottle?** *(tick one box only)*

At most bedtimes, including naps ☐1 Only occasionally at bedtimes, including naps ☐4

At most night bedtimes, but not daytime naps ☐2 Never ☐5 ⟶ ***PLEASE GO TO E4***

At most daytime naps, but not night bedtimes ☐3

E3 **What is usually in the bottle?** *(tick one box only)*

Formula or toddler milk ☐1 Fruit juice/juice drinks ☐4

Expressed breast milk ☐2 Water ☐5

Cow's milk ☐3 Other, please specify……………………….………

E4 **Apart from a bottle, do you feed YOUR CHILD any snacks just before bed (after toothbrushing)?**

Yes ☐1 → *(Please answer question below)* No ☐2

If **yes**, please list what snacks you feed your child in the space provided below

……………………………………………………………………………………………

……………………………………………………………………………………………
